# Contributions of Preventive Social Services in Early Childhood Home Visiting in a Disadvantaged Area of Sweden: The Practice of the Parental Advisor

**DOI:** 10.1177/1049732321994538

**Published:** 2021-02-28

**Authors:** Madelene Barboza, Anneli Marttila, Bo Burström, Asli Kulane

**Affiliations:** 1 Karolinska Institutet, Stockholm, Sweden; 2 Region Stockholm, Stockholm, Sweden

**Keywords:** home visiting, parenting support, early childhood development, nurturing care, social services, constructivist grounded theory, qualitative, Sweden

## Abstract

Early childhood home visiting to improve health and development is commonly delivered by child health care (CHC) whereas home visitors from the social services are rare. We applied a constructivist grounded theory approach to explore the practice and contributions of parental advisors from the preventive social services in a home visiting collaboration with CHC in a socioeconomically disadvantaged area of Sweden. The analysis rendered a conceptual model of a situation-based practice, built on interactive encounters between parents and professionals. It includes strengthening of positive parenting, connecting parents to additional services, early detection of needs and provision of psychosocial support in accordance with each family’s specific situation. Rooted in the training and experience in social work, the practice can be seen as contributory to the delivery of complex support to families through home visiting and could provide input to efforts of improving training of home visitors in different contexts.

## Introduction

### Nurturing Care in Early Childhood

Research from the field of early childhood development (ECD) in the last decade has consistently indicated the importance of ensuring that conditions of nurturing care are in place to enable children to develop to their full potential (Black et al., 2017; Britto et al., 2017; Irwin et al., 2007; World Health Organization et al., 2018). The nurturing care framework encompasses health and nutrition, as well as early learning, security and safety, and responsive caregiving (World Health Organization et al., 2018). Families are understood to need knowledge and resources to develop their capacities to provide nurturing care, and these should be offered through services, programs, and policies in cross-sectoral interventions, using already existing service platforms. Home visiting, center-based parenting programs and parenting groups are among such recommended interventions (Black et al., 2017; Britto et al., 2017; World Health Organization et al., 2018).

### Home Visiting Interventions

Early childhood home visiting has become increasingly common and programs vary widely in terms of goals, target population, length and dosage, delivery staff as well as policy context (Gomby, 2005; Sweet & Appelbaum, 2004). Voluntary in nature, home visiting is often targeted at families who screen positively for “risk-factors” such as low income, young mothers, postnatal depression, drug use, or risk of child maltreatment, for example, in the different models included in the U.S. Federal Maternal, Infant and Early Childhood Home Visiting Program (Duggan et al., 2018). In other countries, for example, the United Kingdom (“Institute of Health Visiting,” 2017) and Sweden (National Guide for Child Health Care, 2019), home visiting is a universal public health service for all children, carried out by child health care (CHC) nurses or public health nurses. Some home visiting programs use social workers as an extra resource for families, giving psychosocial counseling when needed (Goldfeld et al., 2018; “Nurses for Newborns,” 2016), or in a supporting or supervision role to the home visitors (Armstrong et al., 1999; Goldfeld et al., 2018), but most commonly the social services are only involved when child protection issues arise in home visiting.

### Parenting Support in Social Services

Although rarely found in home visiting literature, the social services are more commonly engaged in other parenting support initiatives. Targeting families with children (0–17 years), concern and interest for parenting support has grown in the past couple of decades, and programs for parents have been created and expanded in many countries (Daly, 2015; Daly et al., 2015). In Sweden, a distinct policy area has been developed and was formalized in 2009 through a National Strategy for Developed Parenting Support (Swedish Government, 2010), including access to support for all parents with children of age 0 to 17 years (Daly et al., 2015). Along with formal parenting programs, parenting support run by social services also include parental counseling, lectures, and online resources (Public Health Agency of Sweden, 2013). These activities that fall under the branch of social services are known as preventive social services, in contrast to the social services that exercise public authority, such as child protective services. Preventive social services are staffed by trained social workers and may function within the same premises or separately from the branch that exercises public authority.

Parenting support as a prioritized area within public health and family policy seems to be maintained for the coming years in Sweden. The new National Strategy for Strengthened Parenthood Support identifies the need for both universal and targeted services, through knowledge-based work methods, and the continuous promotion of collaboration between structures and actors (Swedish Government, 2018). Likewise, a recent governmental interim report stresses the importance of continuous strengthening of the preventive social services and the promotion of cross-sectional collaboration with the CHC (SOU, 2018).

### Home Visiting in Collaboration—A New Intervention in Rinkeby, Sweden

Even though parenting support in Sweden is carried out both by the CHC and social services, collaboration in the form of a joint home visiting program, to our knowledge, had not been attempted until 2013 in the area of Rinkeby, Stockholm. The area presents unfavorable socioeconomic and health indicators both for adults and children (Burström et al., 2014; Region Stockholm, 2019) in a context of increasing inequities (SOU, 2017). There is a great cultural diversity in Rinkeby, with 91% of its inhabitants born abroad or by two foreign-born parents, and many families are newly arrived in the country (City of Stockholm, 2020). At the time, the preventive social services received low levels of trust from the community, and few families utilized the existing parenting support services. The CHC, on the other hand—while reaching virtually all families through the National CHC program (Region Stockholm, 2019)—experienced lack of time and resources to meet the large needs of the families in Rinkeby. Both actors wished for a closer collaboration to develop more effective early childhood preventive interventions (Mellblom et al., 2018). A formal extended home visiting program was thus initiated and offered to all first-time parents registered at Rinkeby CHC center. It composed five extra home visits over the child’s first 15 months, in addition to the already existing first visit. The program’s main objectives were to decrease risk factors and improve protective factors influencing the children’s health and well-being and strengthen the parents’ self-efficacy and health (Burstrom et al., 2017). It followed the general content of the National CHC program but with intention to be open for the individual needs of each family. All six visits were made jointly by a team of CHC nurse and a parental advisor from the preventive social services. It was expected that the parental advisors would contribute particularly with support and advice on attachment, stimulus, and interaction between parents and child (Burstrom et al., 2017). During the first program cycle 2013-2016, the professionals gradually developed a content guide for each visit, with themes based on the child’s development phases as well as the common requests from parents, still however with the recommendation to maintain openness to individual needs of families (Mellblom et al., 2018).

The program has achieved high levels of participation where 94% of families who were offered the program’s first phase, 2013-2016, accepted, and 87% of the foreseen home visits were carried out. Since 2017, annual acceptance levels have been above 95%. Parents from more than 30 different countries have participated (Burstrom et al., 2017).

Considering the limited literature on the participation of preventive social services in home visiting, the present study aimed to identify the practice of the parental advisors in the Rinkeby extended home visiting program and explore the possible contributions to the field of early childhood home visiting. The concept of practice—here—is understood as a purposeful, dynamic, and situational interaction with others, involving both theoretical and practical reasoning (Schwandt, 2005, 2014).

## Method

A qualitative study was carried out and included analysis of documentation, interviews with parental advisors, and observation of home visits. The study was developed using a Constructivist Grounded Theory approach (Charmaz, 2006), a method aimed at conducting systematic analysis of social processes and phenomena and developing theory. The method is applied inductively to ground the analysis in the data itself. It involves simultaneous data collection and analysis, constant comparison back and forth between data and data as well as data and categories, during the whole process, and the construction of theory through gradual abstract conceptualization. The researcher aims to gain an insider’s understanding of the research object, and the data are understood to be co-constructed with the participating persons. The method allows for the use of various types of data in the same study (Charmaz, 2006). It was chosen as it provides tools for analyzing data very closely, and it is considered suitable for complex social processes that have not been studied before. The data were collected between September 2013 and March 2016 and from June to October 2019. All authors belong to the research group that has been evaluating the program since 2013 with several published and ongoing studies.

The initial step of analysis regarded the documentation of 481 home visits from 2013 to 2016. The documentation was written by the three parental advisors who were then working in the program, guided by the following questions: “What was my focus during the home visit? How did I act within this focus? What are my thoughts, reflections and impressions after the visit?” It contained extensive descriptions of the parental advisors’ observations of families and subsequent interaction with families during home visits, as well as reflections on their interventions. All documentation was read through and then line-by-line coding was performed on the documentation of 76 visits (16%) whereas reflective memos were written in parallel. Codes were discussed among all authors, and tentative categories were created. The remaining part of the documentation was then analyzed using focused coding, constant comparison, and memo writing. All coding was performed using a computer software. The categories were further developed, and an interview guide was created based on these categories.

Following this, all seven parental advisors from the Rinkeby extended home visiting program, three of whom were working in the first program phase 2013-2016, and four professionals working from 2016 to the present date, were interviewed. The researcher had previously met the four presently active parental advisors during meetings in Rinkeby, while the other three were unknown. The interviews took place at the parental advisors’ office in five cases, at the Karolinska Institute in one case, and at the parental advisor’s home in one case. They lasted 1.5 to 2 hours and were recorded.

All parental advisors were females and, except for one who was a family therapist, they were all trained social workers. They had an average of 16 years (range 7–30 years) of previous experience of social work within different areas of the social services, such as case work, family counseling, school counseling, and institutional care. Within the home visiting program, the parental advisors attended 98 families between 2013 and 2016, and 235 families between 2017 and 2019.

Transcription and focused coding were carried out continuously, allowing for theoretical sampling through adaptation of the interview guide in accordance with progression of the analysis. Sorting, development of theoretical categories, and a conceptual model composed the following steps. Finally, nonparticipant observation of three home visits were made to families who had previously been approached by the professionals and given their consent to participation. Observations were registered through note taking, and subsequently analyzed using focused coding and constant comparison. Saturation of the theoretical categories was achieved at this point. Theory and conceptual model were consolidated and written out in their final version. All steps of the analysis process were regularly shared and discussed between the members of the research team. Preliminary results were presented to the parental advisors and the manager at Rinkeby preventive social services, and their reflections helped shed light on the relationship among categories in the final theory. On various occasions, the preliminary findings were also presented and discussed at seminars for parental advisors and CHC nurses from other areas. The study was approved by the Swedish Ethical Review Authority (Dnr: 2013/877-31/1 and Dnr: 2019-01676), by the CHC center, and preventive social services in Rinkeby. All participating professionals and families had given written informed consent after receiving written and oral information in accordance with the authority’s requirements.

## Results

**Figure 1.**
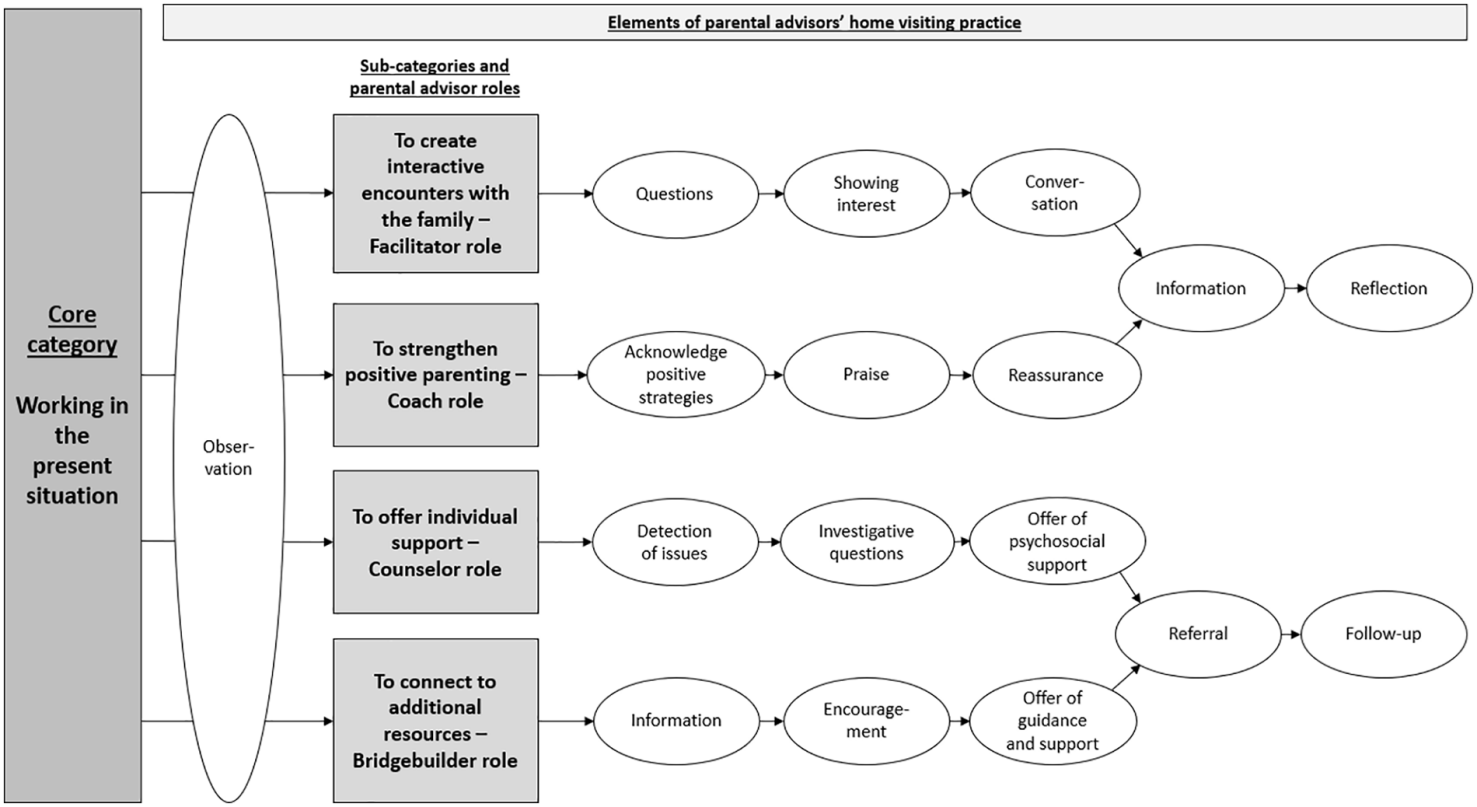
Conceptual model of the parental advisor’s practice during home visits.

The parental advisors’ practice in the home visiting program (illustrated in Figure 1) is characterized in the core category as “Working in the present situation,” and four subcategories composed of elements applied during the home visit. Within this practice, the parental advisors can be understood to assume the four different professional roles of facilitator, coach, counselor, and bridgebuilder, which are tied to the four subcategories.

### Working in the Present Situation

A situation-based approach is central to the parental advisors’ practice, where the intervention is guided by what is going on in the room and the family’s needs and wishes as they are perceived by the parental advisor. When entering the home, the parental advisor initially observes the child’s development, how parents react to and interact with the child, the relationship and collaboration between the parents as well as their interaction with other family members who might be present at the visit:

It is about observing, see what is going on, well I look at these things the whole time. How does the parent hold the child? How do the parents talk to the child? How do they talk about their child?

This initial “reading” of the situation becomes a starting point of the intervention which will guide the parental advisor’s decision on what action should be taken or which content should be introduced. While grounding the intervention in the present moment, the parental advisor delivers content through the three subcategories of *strengthen positive parenting, offer individual support*, and *connect to additional resources.* The fourth subcategory, *create interactive encounters with the family*, becomes a channel through which the other subcategories are delivered.

Working in the present situation thus entails the conscious use of situational input. The parental advisor may ask the parents to tell her about the child’s development and progress. When she notices something relevant in the parents’ stories, or something happening in the room, she picks up on it to introduce a theme or issue into the conversation. The themes come from the Early Child Development (ECD) areas of stimulus, interaction, development, safety, and support networks. The chosen theme may be one that was preplanned for the visit or any other issue that the parental advisor judges to be important for the family at this moment:

And then to acknowledge what you see in the room. You wait until something happens in the room and then you can say, “Do you know how this looks? What great contact you have. It shows on the child what good contact you have,” like that.

Working in the present situation requires that the parental advisor is open and flexible to act instantly on that which might appear and use the pre-established themes as a frame. Any stricter planning of each visit might be more of a hindrance than an enabling aspect of the parental advisor’s work:

I don’t prepare too much but walk into the room and try to be attentive to see what material there is for me to work with in this room that the parent contributes with. If I have my head full of points, then I am not present in the same way. So, I try to be as empty as possible and as present as possible to increase my attentiveness for the parents and catch them in order to see them, understand them.

Acting on what happens in the room at the specific instance renders concreteness to the themes and helps parents relate them to their own actions and experiences.

### Creating Interactive Encounters With the Family—The Facilitator Role

Working in the present situation thus signifies that the parental advisor seeks to meet the family “where they are” and thereby promote a participatory encounter between parents and professionals. An active and supportive dialogue carries the intervention and the parental advisor assumes the role of facilitator by establishing patterns of conversation to keep parents engaged. Different types of questions and expressions of interest are used to get the conversation going around issues that are grounded in the present moment:

Because I believe that if the person you are visiting feels that you are genuinely interested in hearing their thoughts, then I think most people answer to it. [. . .] I think it is about meeting the parents, like, “I am interested in knowing who you are. What do you feel about your child? Tell me! How has it been since the last time we met? What do you notice,” [. . .] All these things are to get the parents interested and I also believe that when they notice that I am interested in them, they will dare to ask those questions.

In this manner, parents’ own questions are stimulated, and information is delivered within the conversation. The parental advisor ensures that the conversation flows back and forth between professionals and parents, with space for talking and listening, questions, interaction, and reflection:

It is ok that it is silent sometimes so that the parent gets the opportunity to reflect on a subject or a question or some information. Then sometimes things awaken in the parents. Also, that there is time to ask. For questions to be asked there needs to be time. We can show that in different ways. We can ask if they have any questions. You can also make space for it by leaving silence.

The meeting builds on inclusiveness. The parental advisor therefore gives specific attention to the more silent ones, often fathers, or those with language barriers. Care is taken to check that the information delivered has actually been understood, and to ensure that the intervention makes sense and is useful to the parents.

A main challenge to establish an interactive encounter is to be accepted and trusted by parents. Most parents have never met the parental advisor before the first home visit, and while the nurse’s tasks are clear, the parental advisor’s role is usually not as obvious to parents. In addition, the parental advisor carries an organizational identity which is more complicated, and which may cause initial suspicion from the parents. She is aware of a common understanding among families that the social services only make home visits to families where there are problems and that the parents may believe that the parental advisor comes from the child protective services, and not the preventive social services. The parental advisor therefore usually avoids presenting herself as coming from the social services in an effort to gain parents’ acceptance:

We don’t say, “Hello, I am A and I come from the social service,” but we say, “I am a parental advisor.” And most of the time there is no conversation around this, and I guess some parents understand and others don’t. I don’t think so many understand at the first visit. And when they then understand it later, we have already established a relation and there is kind of no big deal.

However, how to deal with the “social service identity” is not a simple issue for all parental advisors. Some of them are not completely comfortable with this practice and argue that ideally, the social services should be clearly introduced in an attempt to promote a positive relationship between families and the institution as well.

### To Strengthen Positive Parenting—The Coach Role

Affirming and strengthening parenting skills is one of the core actions in the intervention. The parental advisor aims at making parents aware of all positive strategies they already know and do by pointing them out when she observes them during the visit and reaffirming them during the conversation:

I would, if there is a good moment, if the child is very satisfied and lying in the parent’s arms, I could put words on it: “Oh how good she feels when she is lying there so close to you,” or something like that.

The parental advisor can here be portrayed as a coach who supports each family to maximize their potential. To maintain a light and positive tone during the home visit is important, and the parental advisor uses strengthening comments and praise plentifully in reaction to what parents say and do. But, it is also important to acknowledge that parenting can be hard at times. It is reassuring for parents to hear that the parental advisor understands how tough it might be and that it is normal to experience challenges. To affirm and strengthen positive parenting is also a good universal strategy which is useful for all parents, even those families where everything is working smoothly, and parents seem to need very little support and advice:

Well we all need someone who sees us. And as a new parent, even if you are totally super, as if you haven’t done anything else in your life, it feels just as good to hear that you do a fine job as a parent. Like the one who struggles a bit also feels good to hear. We all need reassurance.

In her role as coach, the parental advisor also aims at demystifying issues that are often difficult to discuss, such as relational problems or mental health issues. She attempts to show that it is ok to talk about sensitive subjects such as violence and genital mutilation, that it is part of the universal prevention agenda within the program in the same way as child health or feeding:

I have a standard thing that I say when we talk about something that we know might be sensitive. Then I say, “we talk to all parents about this,” and I kind of, “We don’t think that you would hit your child, for example. We think that this is so important that we think we need to talk to all parents about it.” I try to talk on a general level so it doesn’t become personal for the parent, but rather, “we know that it can be like this,” for example.

The parental advisor finds it important to help parents themselves reflect on how they feel and think about issues, reach their own decisions, and take responsibility for their own parenting process. Asking questions that help parents reflect is a step in building parental ownership.

### To Offer Individual Support—The Counselor Role

In addition to acknowledging existing positive parenting practices, the parental advisor also focuses on specific needs or problems in families. These might be taken up by the parents themselves or through the parental advisor’s observations during the home visit. When she detects signs of things that may not work so well, she uses explorative questions to investigate further and also shares her observations with the family:

And to notice and pick up something that you hear, and I guess this is where the sensitivity comes in, that I can sense an issue, then I can pick it up and put light on it. “I hear that you are having a tough time and that he is sleeping badly at night. How do you deal with that? Do you have a strategy? Who helps you? Do you have any support,” [. . .] And recognize that this is a risk factor for coping.

If the parents also recognize the need, the parental advisor may suggest they deal with it during the visit. She can then deliver psychosocial support, drawing from her experience and training in family counseling. Alternatively, she may offer to book an extra session at her office to work further on the issue. When there is need, she will make necessary referrals to more specialized services. These specific needs or problems are then followed up during the coming home visits.

As it is most commonly the parental advisor who suggests the extra support, she is aware that care needs to be taken to ensure that the voluntary aspect is clear to the parents. At the same time, she also recognizes that her own proactivity is an important aspect when offering this type of support:

There can be things that complicate the families’ lives quite a lot, and then we can just say, “We can see that it is tough for you right now. Shall we book a time pretty soon and we will come back to check how things are going?” And we can say that straight out, openly to the families and basically every time there are no problems, on the contrary, they are rather thankful that we took time to look after them in some way.

In addition to early detection of extra needs and fast counseling support, the parental advisor also aims at creating a channel to the preventive social services. She informs of counseling and parenting support programs that can be accessed until the child is 18 years old. This has resulted in some cases where families have reached out to the parental advisor in later situations of need. Nevertheless, no significant increase in demand by parents outside of the home visits or after the completion of the program has yet been noted by the parental advisors.

### To Connect to Additional Resources—The Bridgebuilder Role

The parental advisor also acts to establish a link to different levels of the Swedish public service system for the parents in the program, many of whom are fairly new in the country. During the first home visits, the parental advisor introduces the local open daycare to all families. It is considered an important resource for play and strengthening of parenting skills as well as social networking. Its physical location next to the preventive social services enables a close collaboration with the open daycare staff with the possibility to follow-up if parents turn up to the activities. When the parental advisor perceives that information and encouragement are not enough for isolated parents to dare make a visit, she will offer to accompany them and introduce them to the staff and other parents:

The feeling of being able to help or supply a contact. To be able to say, “We know a place where you can go, where you can meet other people,” [. . .] The men are not always so present at home, so the women are quite often alone. And then when we can tell them about this place called open daycare, where you can go.

As a bridgebuilder, the parental advisor is required to inform about municipal and regional services that are relevant to families, and she receives requests from parents on issues such as how to access language classes, the social security system, or child care. She will help opening doors and create channels, and by observing each family’s specific situation, she decides how much support she needs to offer. Encouraging parents to do as much as possible on their own is considered important:

I can indicate, and then I need to think about how I need to indicate? Do I need to accompany someone somewhere or is it enough to tell someone, “you can phone this place?”

Still, some families may need both motivation and support from the parental advisor to overcome thresholds and take new steps:

Well, when I meet a parent who, for example, has problems in relation to the child’s father who has a problem with addiction, then I can connect to the addiction clinic if the parents want to. And I ask: “Would you think it a good idea if we meet together with the addiction clinic so they can inform what support is available to families?” I mean it is easy for me to do these things, it is not hard, and I think of what benefits the family.

The parental advisor also acts as a guide in the complex system of social services, which have low levels of trust in the area. The social services are perceived as being called when families have problems and there is a fear of child protective services who may take away the children, and thus such contacts may be stigmatizing for parents. The parental advisor is aware of this and works to facilitate contacts to relevant areas on parents’ request, and she can refer to specialized help when parents need and wish:

I believe it is beneficial to have a long experience from the social services. To have a broad competence I think is useful as we channel a lot of things to other contexts and so on.

Although not part of the branch that exercises public authority, the parental advisor also has reporting duty in cases of concern to a child’s welfare. This, however, is not perceived as a barrier in her work in the home visits. On the rare occasions when the parental advisor reports a case, she attempts to keep transparency by sharing information with the family and act as a support when there is need:

If I report a case of concern to the social services, I very much want to participate in the visit if possible and make the transition so that the parents feel that they receive help.

Despite aiming to open doors and lower thresholds, the parental advisors do experience a barrier regarding housing issues. Many families have precarious and unstable residence situations as a result of the general housing shortage and subsequent high prices in the Stockholm region. While recognizing the importance of adequate living conditions for the children’s health and well-being, the parental advisor has very limited possibilities of helping the families. She can supply information and refer to housing services, but with the knowledge that many of the families find that their only option is insecure contracts on the informal housing market.

## Discussion

### A Practice to Meet the Family Where They Are

The parental advisors in Rinkeby have developed a situation-based practice which composed the facilitation of an interactive meeting where the main foci are to strengthen positive parenting, offer psychosocial support when needed, and connect families to additional services in the community. The choice of action is determined by the continuous observation of the family in the present situation and is a response to the needs and wishes of the family as they are perceived by the parental advisor.

Professionals’ effort to meet the family where they are can similarly be found in other settings of care. For example, Davies et al. (2017), studying best practice of provider–parent interaction in hospitals, presented what they call “exquisitely attuning to particularities of the situation in the present moment” (p. 417). This involves that the provider listens, observes, discerns, interprets, and acts according to what is most important to the parents at the particular moment. This aspect of practice seems to correspond well to the main category found in the conceptual model from the Rinkeby home visiting study.

Within the home visiting literature, this type of practice is identified by McDonald et al.’s (2012) as a home visiting approach which is responsive and tailored to parents’ needs, rather than manual-based where content and procedures are to be followed with a high degree of fidelity. According to the authors, the application of this responsive approach depends on the experience and skills of the home visitor, rather than on program-specific training (McDonald et al., 2012).

### The Challenges of Complexity

A responsive practice consequently demands flexibility and preparedness by the home visitor to take on different roles in their interaction with parents. In the roles of facilitator, coach, bridgebuilder, and counselor, the parental advisors have developed a home visiting intervention that spans over promotion, prevention, treatment, and referral. They are primarily supported in these roles by their professional training and experience within social work, counseling, therapy, and process facilitation, as well as the professional location within social services. Although many other home visiting program curricula present a similar complexity, several studies have also indicated that home visitors commonly face challenges that can be related to lack of training, skills, and experience.

An example of this is how the challenges of complexity have been given attention in several articles on British health visiting (Bidmead et al., 2015; Cowley et al., 2015; Malone et al., 2016). Home visiting is here described both as a “helping process” with a utilitarian objective (Bidmead et al., 2015), but also containing moments when the professional is required to establish a therapeutic relationship when parents encounter challenging situations (Bidmead et al., 2015; Cowley et al., 2015). Malone et al. (2016) discuss the challenge faced by health visitors when applying this practice with a diversity of families in a universal context. They conclude that professionals need to develop new values and attitudes that are adequate both for a health promoting practice, as well as more sophisticated assessment and communication skills, and that health visiting requires more complex skills than what is currently acquired through health visitors’ training (Malone et al., 2016).

### Dealing With Adversities

Although universal early childhood home visiting was new to the parental advisors in Rinkeby, to observe possible adversities or special needs and attend to these through psychosocial support was already central to their general professional practice within the preventive social services. This capacity, brought into home visiting, may be considered a contribution to increased complexity in the offer of support to families.

Studies from other contexts have outlined home visitors’ difficulties in identifying and addressing adversities such as intimate partner violence, mental health, and substance use, and subsequently related it to lack of training (Duggan et al., 2018; West et al., 2018). Jack et al. (2017) found that to address intimate partner violence, home visitors need knowledge and skills to use flexible strategies adapted to each specific family and context, rather than screening tools. Similarly, Albaek et al. (2018), in a metasynthesis of professionals’ experiences in exploring and identifying adverse childhood experiences, reported a perceived lack of both competence and means. In addition to the studied professionals’ request for knowledge, experience, training, support, and guidelines, the authors also pointed to the need to consider the complexity of cases and professionals’ psychological distress, rather than providing simplified tools such as guidelines and screening protocols (Albaek et al., 2018). Still, the capacity for early detection and support is of main importance in home visiting as it may prevent a worsening of the parents’ situation, increase the likelihood of disclosure of deeper adversities or needs and facilitate referral to specialist services (Cowley et al., 2015).

### Referrals to the Service Network

Home visitors’ role as service coordinators has been discussed by Goldberg et al. (2018), who argue that this should be considered a central and complex activity that goes beyond simply connecting families with services, but also involves strengthening families’ capacity to navigate the service system, as well as keeping them engaged once they have accessed the service. The authors further mention that this activity, in itself, may improve parents’ problem-solving capacity and increase persistence. Still, they found that service coordination holds a marginal place in most home visiting programs (Goldberg et al., 2018). These findings reinforce the relevance of the parental advisors’ bridgebuilder role, which is carried out continuously over the program duration with dose and intensity according to each family’s current needs. Specific also to the parental advisors, is how their long experience in and knowledge of the social services become an asset when parents need to access such services. However, it cannot be understated that successful referrals also depend on the existence of a working service network and structural resources, which are scarce in many home visiting contexts. The case of housing problems in Rinkeby is a telling example where the parental advisors, despite time and competence, perceive a very limited capacity to help.

### Facilitating the Encounter With Families

An interactive encounter between parents and professionals carries the home visiting intervention in Rinkeby and the parental advisor works systematically to promote this encounter using facilitator skills and techniques. The importance of this type of skill is recognized in the parenting support literature in terms of communication skills (Bidmead et al., 2015; Blue-Banning et al., 2004; Schultz et al., 2019; West et al., 2018) or process delivery skills (Moore et al., 2012). Davies et al. (2017) outline how skills in engaging authentically with families through a set of connecting behaviors are part of best practices in provider–parent interaction. These include listening, empathizing, clarifying and verifying understanding, focusing on the positive, and pacing the interaction to parents’ needs (Davies et al., 2017). This description seems to come close also to how the parental advisors act in the meeting with parents. Home visiting studies have further indicated that these kinds of facilitation or relationship skills are developed from a combination of theoretical study and practical experience (Duggan et al., 2018; Stern, 2008) and to acquire them takes considerable time (Malone et al., 2016).

### Parental Engagement and the Preventive Social Services

In addition to the parental advisor’s facilitation competence, the successful establishment of an interactive encounter also centrally depends on the parents’ actual engagement. Engagement is understood as being collaboratively developed between professionals and parents (Duggan et al., 2018; Korfmacher et al., 2008), which indicates the need to establish a positive relationship. The findings indicate that the parental advisors are concerned that being a representative of the preventive social services may cause hesitancy in the parents. This reaction was also noted in a recent study on Danish maternity care services, where parents in vulnerable positions experienced fear of possible consequences of accepting services and disclosing sensitive information (Frederiksen et al., 2021). The parental advisors, however, do not generally perceive any difficulty in establishing good contact once the initial acceptance is achieved. Neither is the mandatory reporting duty perceived as a hindrance, contrary to what was found in a study of child abuse prevention in home visiting (Matone et al., 2018). The high levels of participation as well as very high levels of satisfaction shown in surveys with parents from the program (Burstrom et al., 2017) seem to reinforce the perceptions that a positive relationship is created. Still, the home visiting program has not yet led to an increase in demand from families for other parenting support resources within the preventive social services. Speculations can be made as to whether parents are unaware of or skeptical toward the preventive social services despite good contact with parental advisors, indicating that their relationship may be only with the parental advisor and not associated with the service as a whole. It may also be that existing services do not meet parents’ actual needs, or perhaps parents simply do not feel the need for additional parental support or seek it elsewhere. These are aspects with potential for further studies into parents’ perception of need and understanding of the role of the preventive social services, which could be a relevant contribution to the development of home visiting and parenting support.

### Strengths and Limitations

Although not the focus of this study, it is important to also place the parental advisors’ work method into its immediate context of the home visiting program, carried out in a team of two professionals. The exercise to separate this specific process was made for study purposes only and hence provides a limited understanding of the full intervention. However, the collaboration between the parental advisors and CHC nurses in Rinkeby has already been investigated in an earlier evaluation of the program (Burstrom et al., 2017), where both groups of professionals strongly confirm the value of this teamwork. They recognize how their professional competencies and expertise complement each other and form a totality that is new in terms of parenting support, both for the CHC and preventive social services.

With regard to methods, the documentation of 481 home visits provided detailed descriptions that enabled observation of a wide range of aspects in terms of child’s age, family composition, situation, and parental concerns. However, they only covered the practice of three parental advisors. The interviews therefore served to compare and investigate the practice of all professionals working in the two different phases of the program. This comparison indicated a large degree of homogeneity in all parental advisors’ descriptions of practice. The nonparticipant observations provided an additional source of data where the researcher could experience the parental advisors’ work in real life and verify that the theoretical categories and conceptual model seemed to be relevant and well-integrated. Three visits proved sufficient for this purpose, and no data were collected that influenced, changed, or further developed the theory. It was thus decided that it was not justified to request that more families open their homes for home visit observations. The member checks of results provided for final assurance of the theory’s completion.

An important consideration to be made regarding the fact that all parental advisors were of Swedish origin, working in a multicultural setting. The parental advisors were therefore asked in the interviews to reflect and discuss this from their own understanding. These reflections showed awareness of how cultural background may influence both understanding and actions. At the same time, a main reason choosing to work in Rinkeby was the interest in cultural diversity and seeing this as an opportunity for professional development. It was also pointed out that culture can be understood in many ways, not only through nationality, and the parental advisors portrayed working in Rinkeby as an exercise of discovering the many facets of diversity and commonality as they are present in each meeting between individual families and professionals. Still, this study did not consider the parents’ perspectives; nevertheless, the recommendation is made to develop further research into this area.

The context of Rinkeby, with its large cultural diversity, may however have an influence on the transferability of findings. It seems reasonable to believe that the part of practice related to introducing and assisting access to other services and resources in society may have less prominence or be composed in different ways in areas with different population characteristics. It would therefore be valuable to investigate how well the theory applies in the scale up of the program to other areas.

## Conclusion

The findings from this study showed how the parental advisors from preventive social services have developed a home visiting practice, founded on their social work training and experience, containing aspects that are assessed as relevant to ensure healthy ECD in a nurturing care environment. Several of these aspects have presented a number of challenges to home visitors from other professional background and experiences, such as early detection of adversities and needs, and offering of initial psychosocial support to families within the home visiting environment. The centrality of connecting and referring to additional community services, also encompassing the institutional connection to social services, was further observed to be a strength in the intervention. This study thus indicates that the parental advisors’ practice may be contributory to home visitor capacity to deliver complex supportive actions to families through postnatal home visiting. It appears that this practice, rooted in the tradition of social work, could be a valuable contribution to consider in efforts to improve home visitor training in different contexts.
